# The association of left atrial volume with age, ethnicity and cardiovascular risk factors in men and women: the Multi-Ethnic Study of Atherosclerosis (MESA)

**DOI:** 10.1186/1532-429X-18-S1-O65

**Published:** 2016-01-27

**Authors:** Filip Zemrak, Bharath Ambale Venkatesh, Gaby Captur, Jonathan Chrispin, Elzbieta H Chamera, Mohammadali Habibi, Saman Nazarian, Saidi A Mohiddin, James Moon, Steffen E Petersen, Joao A Lima, David A Bluemke

**Affiliations:** 1 grid.4868.20000000121711133Centre of Advanced Cardiovascular Imaging, Queen Mary University of London, London, UK; 2 grid.411935.b0000000121922723Cardiology Division, Department of Medicine, Johns Hopkins Hospital, Baltimore, MD USA; 3 Cardiovascular Imaging, Barts Heart Centre, London, UK; 4 grid.94365.3d0000000122975165Radiology and Imaging Sciences, National Institutes of Health, Bethesda, MD USA

## Background

There are limited data assessing the association of demographics and cardiovascular risk factors with left atrial (LA) dimensions measured by cardiovascular magnetic resonance (CMR). The aim of this study was to determine the association of LA volume with gender, demographic factors, cardiac structure and cardiovascular risk factors.

## Methods

LA volume indexed to body surface area (LAVi) was measured by CMR using steady-state free precession cine long and short axis images in 2576 participants of the Multi-Ethnic Study of Atherosclerosis (68.7 years, 53.0% women). We used gender stratified regression models to evaluate the association of LAVi as the dependent variable with demographic and cardiovascular risk factors, left ventricular (LV) parameters and diagnosis of coronary heart disease as independent variables. LAVi between ethnicities were compared using analysis of variance (ANOVA) with Tukey's post-hoc analysis. To determine normal LA dimensions we also selected a group of participants with normal body mass index (≥18.5 and < 25 kg/m^2^), without hypertension, diabetes, coronary heart disease, congestive heart failure, LV systolic dysfunction (defined as ejection fraction less than 50%), LV hypertrophy or atrial fibrillation (n = 285, 65.6 years, 61.8% women).

## Results

The unadjusted mean LA volume index in the whole cohort was 36.5 ± 11.4 ml/m^2^ and was 9% smaller in men (35.9 ± 11.1 vs. 37.0 ± 11.6 ml/m^2^, p < 0.05). LAVi was greater with age in men (β = 0.2 ml/m^2^/yr, p < 0.0001) and women (β = 0.3 ml/m^2^/yr, p < 0.0001). Both Chinese American men and women had significantly (p < 0.05) smaller LAVi compared to other ethnicities (Figure 2). History of coronary disease was associated with 10% larger LAVi in women (β=3.7 ml/m^2^, p < 0.05), but not in men (p = ns). In the normal reference cohort free of cardiovascular disease there were no differences in LAVi by gender (men 34.5 ± 9.9 ml/m^2^, women 36.0 ± 10.2 ml/m^2^, p = 0.30).

**Figure 1:**
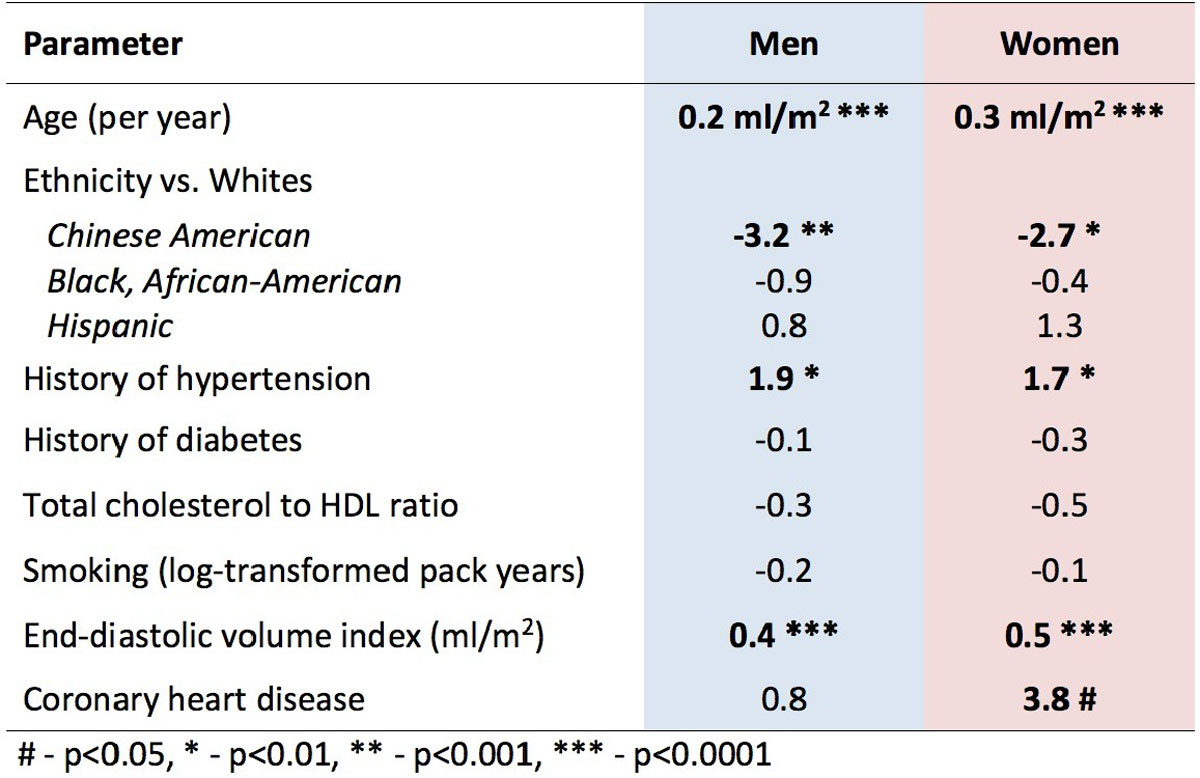
**Multivariable linear regression models showing associations of left atrial volume index in the full cohort with cardiovascular risk factors**.

**Figure 2:**
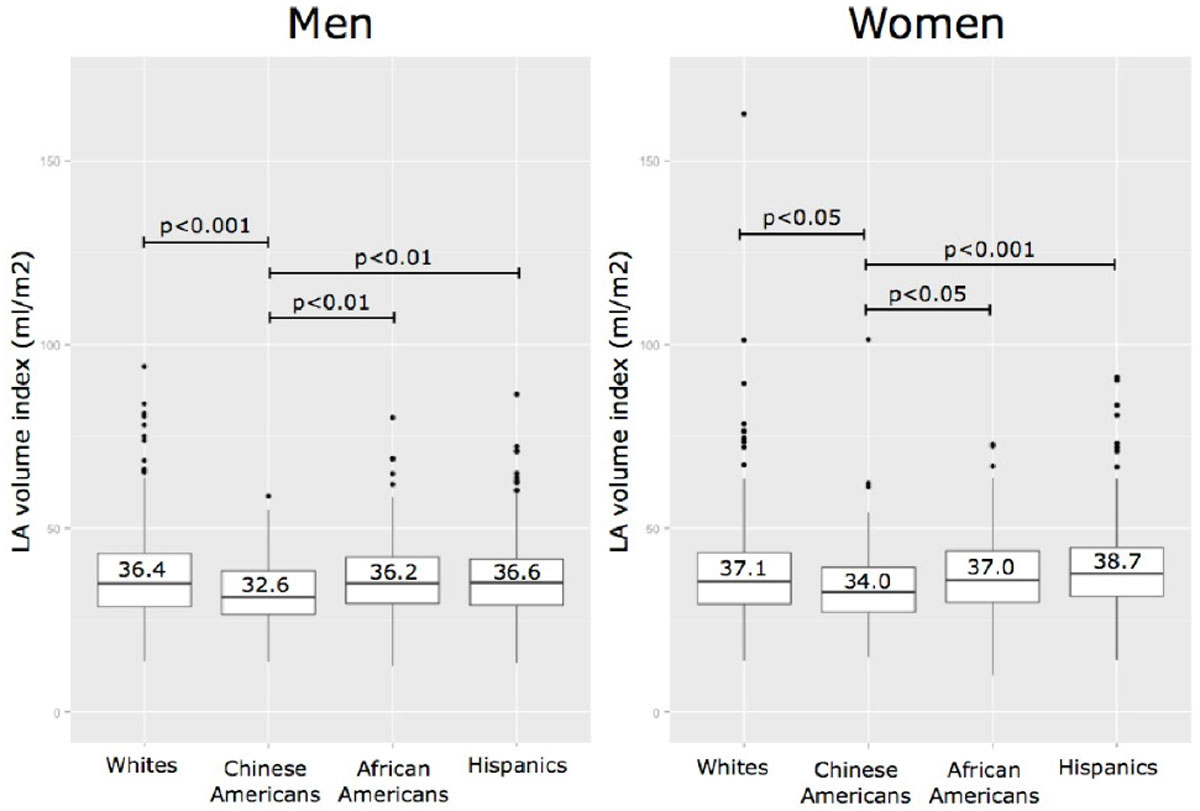
**Chinese American men and women had smaller left atrial volume index**. Boxes represent the interquartile range (IQR) and whiskers are within 1.5 *IQR, outliers are plotted as points, the line within the box represents the median, mean values presented as number, p-values of Tukey's test.

## Conclusions

LAVi enlargement in response to age and cardiovascular diseases is different in men and women, and may be also influenced by ethnicity. However, gender did not influence LAVi in the "normal" reference cohort free of cardiovascular disease.

